# Present and Future Research on Anal Squamous Cell Carcinoma

**DOI:** 10.3390/cancers13153895

**Published:** 2021-08-02

**Authors:** Laurie Spehner, Jihane Boustani, Luc Cabel, Jérôme Doyen, Angélique Vienot, Christophe Borg, Stefano Kim

**Affiliations:** 1 Interactions Greffon-Hôte-Tumeur/Ingénierie Cellulaire et Génique Research Unit INSERM UMR1098, University of Bourgogne Franche-Comté, 25020 Besançon, France; laurie.spehner@efs.sante.fr (L.S.); a3vienot@chu-besancon.fr (A.V.); christophe.borg@efs.fr (C.B.); 2 Department of Medical Oncology, University Hospital of Besançon, 25030 Besançon, France; 3 Department of Radiotherapy, University Hospital of Besançon, 25030 Besançon, France; jboustani@chu-besancon.fr; 4 Department of Medical Oncology, Curie Institute, 75005 Paris, France; luc.cabel@curie.fr; 5 Department of Medical Oncology, Centre Antoine-Lacassagne, 06189 Nice, France; Jerome.DOYEN@nice.unicancer.fr; 6 Clinical Investigational Center, INSERM CIC-1431, Centre Hospitalier Universitaire de Besançon, 25030 Besançon, France; 7 Department of Oncology and Radiotherapy, Nord Franche Comté Hospital, 25209 Montbéliard, France

**Keywords:** anal carcinoma, research, trials, immunotherapy, chemotherapy, biomarkers

## Abstract

**Simple Summary:**

This is an exciting moment in clinical research in the squamous cell carcinoma of the anus (SCCA). Historical barriers regarding this disease are vanishing thanks to the creation of research networks in this rare pathology, gained knowledge in tumor biology and its environment in this human papillomavirus (HPV)-induced disease in more than 90% of patients, and the arrival of taxane-based chemotherapy regimens as well as immunotherapies and its novel combinations. This review sheds light on the present and the future of research on SCCA, new understanding of its rational for ongoing clinical trials, with special focus in locally advanced and metastatic diseases.

**Abstract:**

Squamous cell carcinoma of the anus is an orphan disease, and after more than three decades of no substantial advances in disease knowledge and treatment, it is finally gaining momentum with the arrival of a taxane-based chemotherapy and immunotherapy. Currently, about 20 combination clinical trials with an anti-PD1/L1 are ongoing in localized and advanced stages, in association with radiotherapy, chemotherapy, tumor vaccines, anti-CTLA4, anti-EGFR, or antiangiogenic molecules. Moreover, a new biomarker with high sensitivity and specificity such as HPV circulating tumor DNA (HPV ctDNA) by liquid biopsy, is improving not only the prognostic measurement but also the treatment strategy guidance for this disease. Finally, better understanding of potential targets is reshaping the present and future clinical research in this unique, HPV genotype-16-related disease in the great majority of patients.

## 1. Introduction

Even though the squamous cell carcinoma of the anus (SCCA) is still considered a rare disease, its incidence is steadily increasing worldwide [1], in particular in the advanced stage [2]. Ninety percent of cases are related to HPV infection, mostly genotype 16, and HIV positive patients are especially at high risk [3].

About 85% of patients are diagnosed at a localized stage [4]. For these patients, chemoradiotherapy (CRT) is the standard of care, and it induces a complete response in 85% of patients [5,6,7]. Salvage surgery is indicated in case of residual disease or local progression. However, about a third of patients experience recurrence with a progression-free survival (PFS) rate at 5 years less than 70% based on phase III trials. Moreover, most patients included in these trials assessing chemoradiotherapy were at an early stage (I and II), and the recurrence rate is even higher in patients with a locally advanced disease. In fact, the 5-year PFS rate was 47% when the tumor was greater than 5 cm, and 35% in case of nodal involvement, and even lower when both factors were present [8]. Even though more recent data suggest that newer imaging modalities can increase the detection of small nodal involvement, a process known as nodal stage migration with consequent improvement of prognosis (Will Rogers phenomenon) [9], the increased relative mortality in the last years is in favor of a real rise of more advanced diseases at diagnosis [2]. Then, a more aggressive personalized approach seems mandatory for patients with locally advanced disease.

In the metastatic setting, two recent prospective phase II trials settled a new standard in this disease [10,11]. First, Epitopes-HPV02 was a multicenter confirmatory phase II trial of the high efficacy of the combination of docetaxel, cisplatin, and 5-fluorouracil (DCF) observed in the Epitopes-HPV01 trial [12,13]. The PFS rate at 1 year was 47%, with an objective response rate (ORR) of 89% including 45% of complete responders [10]. The modified DCF regimen (mDCF) was far better tolerated (53% vs. 83% of grade III/IV toxicity rate) with no febrile neutropenia by contrast to standard DCF regimen with a similar efficacy. mDCF became the first prospectively validated standard regimen in metastatic SCCA [14,15]. Recently, a pooled analysis of updated data of 115 patients in Epitopes-HPV01 and 02 trials demonstrated a long-lasting complete response in 25% of patients at 5 years, with an overall survival rate of 44% after DCF [16]. Second, the InterAACT “pick the winner” randomized phase II trial evaluated two doublet regimens: carboplatin plus paclitaxel (CP), and cisplatin plus 5-fluorouracil (CP). Both regimens had similar efficacy (ORR of 59% and 57% including 13% and 17% of complete responses, respectively) and toxicity profiles (grade III/IV toxicity rate of 71 and 76%, respectively) and failed to demonstrate its prespecified main endpoints [11]. However, CP regimen had lower severe-adverse events and a trend for improved overall survival and was considered as better regimen than CF.

Then, in chemorefractory patients, anti-PD1 immunotherapies demonstrated efficacy in a subgroup of patients [17,18,19,20], and several combination trials are ongoing in second-line, as well as in first-line setting in association with mDCF or CP [21,22].

Moreover, deeper understanding of the biology of this cancer and the rise of new biotechnologies allowed the emergence of not only new biomarkers such as HPV ctDNA [23] but also potential new therapeutic targets [24,25].

In this review, we describe the recent progress and ongoing clinical trials in SCCA.

## 2. Localized Disease: What’s Next?

Since encouraging results of chemoradiotherapy (CRT) first published by Nigro and colleagues’ in 1974 [26], definitive CRT with mitomycin (MMC) and 5-fluorouracil (5FU) have progressively replaced the surgical abdominoperineal resection and have become the standard of care for localized disease, allowing to preserve the organ function and to improve the local control for the majority of patients [27]. In the last four decades, all phase III trials confirmed CRT (delivering 60 Gy on the involved tumor fields) with MMC and 5FU as the standard regimen with no clear advances in localized SCCA [5,6,7,28,29]. However, some minor changes have been recommended based on phase II trials such as (i) replacement of infusionnel 5FU by oral capecitabine [30,31], (ii) more conformal technique such as intensity-modulated radiotherapy (IMRT) to spare the organs at risk [32,33], and (iii) de-escalation of prophylactic radiotherapy dose to 36 Gy to reduce toxicity [34,35]. Several trials are currently ongoing to evaluate the de-escalation or dose escalation in localized disease depending on the tumor size at diagnosis [36]. PLATO (PersonaLising Anal cancer radioTherapy dOse) is a single protocol platform, comprising three separate trials (ACT3, ACT4, and ACT5) with the aim of personalizing radiotherapy dose from early to locally advanced disease [37]. The ACT3 trial is a prospective non-randomized phase II trial that enrolls patients with T1N0 anal margin tumors who have undergone local excision. Patients with surgical margins ≤1 mm receive an additional lower-dose CRT (41.4 Gy in 23 fractions) with the aim of demonstrating a 3-year loco-regional failure rate of <10%. The ACT4 trial is a randomized phase II trial that compares a standard-dose CRT (50.4 Gy in 28 fractions) with a reduced-dose CRT (41.4 Gy in 23 fractions) in patients with T1, T2 <4 cm N0 disease, with the aim of demonstrating an acceptable low rate of loco-regional failure rate while reducing toxicity. The ACT5 trial is a randomized pilot, phase II, and phase III trial that compares standard-dose CRT (53.2 Gy in 28 fractions) with two higher doses of CRT (58.8 Gy and 61.6 Gy, both in 28 fractions), in patients with locally advanced anal cancer (T3, T4 any N, or T2N2-3) with the aim of demonstrating a significant reduction in loco-regional failure with an acceptable toxicity.

Anti-PD-1/PD-L1 immunotherapies are promising in localized disease. Preclinical data indicate that antitumor efficacy is increased when anti-PD-1/L1s are combined with radiotherapy. A durable expression of PD-L1 on tumor cells surface was observed after radiotherapy and was correlated with a decrease in Myeloid-derived suppressor cells (MDSC), and better survival in mice treated with an anti-PD-L1 antibody [38,39,40]. In addition, radiotherapy elicits tumor antigen release and modulates the tumor cell phenotype, leading to both an activation of immune responses and an increase in immune recognition. Irradiated tumors release danger signals that trigger dendritic cells (DC) activation, as well as a dose-dependent increase in MHC class I presentation leading to tumor recognition, resulting in a higher anti-tumor CD8-T lymphocyte infiltration and cytotoxicity. These phenomena, along with the increase in expression of PD-L1 ligands in tumor cells, could enhance the efficacy of checkpoint inhibitors not only at irradiated field but also in distant tumors located outside of the radiation field known as “abscopal effect” [41,42,43]. Hence, immunotherapy could be synergic to CRT to improve the control of local disease, as well as distant micrometastases, leading to a better recurrence-free survival and cure. The addition of an anti-PD1/L1 immunotherapy to CRT has already demonstrated efficacy in PD-L1+ stage III non-small-cell lung cancer patients [44], and more recently in stage II-III esophageal cancer [45]. Interestingly, in both trials, local and distant progression were reduced with the addition of an anti-PD1/L1 immunotherapy. In anal carcinoma, the interest of a concomitant and/or adjuvant immunotherapy with an anti-PD1/L1 or an HPV tumor vaccine (ADX11-001) is currently being evaluated (Table 1) [22].

## 3. Locally Advanced Disease: Time to Shift the Paradigm?

CRT is the standard of care in locally advanced stage III disease [5,6,7]. The lack of an effective concurrent molecule [46], the failure of higher doses of radiotherapy [7], and the absence of an effective neoadjuvant/adjuvant chemotherapy regimen [5,7], halted the implementation of more aggressive strategies for locally advanced disease. Anti-EGFR antibodies are validated in cancer treatments, including localized head and neck cancer in combination with radiotherapy [47]. However, the addition of cetuximab to CRT in SCCA patients was unacceptably toxic [48,49]. Panitumumab, a fully humanized anti-EGFR, was evaluated in association to standard CRT according to the doses previously defined in a phase I trial [50]. Despite its acceptable safety profile, the complete response rate at 8 weeks from CRT was lower than expected, and unmet to pursue to a phase III trial. ACCORD03 trial evaluated the interest of a higher radiation dose (>60 Gy) for tumors greater than 4 cm with nodal involvement. Unfortunately, it failed to improve outcomes such as recurrence-free survival and colostomy-free survival [7]. ACCORD03 and ACT II trials also evaluated the interest of an induction or a maintenance chemotherapy with cisplatin and 5FU (CF), respectively. Both trials were negative. However, CF regimen is not a good candidate in localized disease with significantly worse DFS and OS than MMC-5FU in association with radiotherapy [6] (Table 2). Thus, it is legitimate to consider that the failure of the neoadjuvant/adjuvant strategy was related to the ineffectiveness of the chemotherapy regimen more than the strategy itself. Nevertheless, DCF regimen could overcome this problem. In Epitopes-HPV02 study, a subgroup of 16 patients with synchronous metastases previously untreated, all patients presented an objective response after DCF with 56% of complete response of all involved sites including primary tumor [10]. The pooled analysis of Epitope-HPV01 and Epitopes-HPV02 studies confirmed these data [16]. None of the 29 patients with synchronous metastases presented a disease-progression at first evaluation during DCF administration, and the ORR was reached in 26 (89.7%) patients, including 16 (55.2%) patients with a complete response. In addition, in the whole Epitopes-HPV population of 115 patients treated with DCF, 25% of patients were still alive and disease-free at 5 years. Along with the high complete response rate, these findings suggest the potential use of DCF in neoadjuvant/adjuvant setting [16].

Anti-PD1/L1 antibodies are also potential candidates since long-lasting complete responses have been seen in chemorefractory patients in advanced SCCA [51]. Moreover, immune biomarker data of Epitopes-HPV01 and -HPV02 trials showed that DCF is a good backbone chemotherapy to combine with anti-PD1/L1 since it increases antitumor human telomerase (hTert) immunity and decreases MDSC, two major factors significantly correlated with prognosis [10,24]. The association of DCF and an anti-PD1/L1 is feasible, with no particular safety signal in SCARCE trial in the metastatic setting, which evaluated DCF with or without atezolizumab [21]. The phase II INTERACT-ION is now ongoing to evaluate the efficacy and safety of DCF with ezabenlimab, an anti-PD1 antibody, as neoadjuvant treatment in stage III SCCA patients treated with CRT (NCT04719988). Other ongoing trials aim to evaluate the clinical response and toxicity of CRT with immunotherapy either in the adjuvant setting alone (EA2165 with Nivolumab; NCT03233711) or in both, the concurrent and the adjuvant setting, in RADIANCE trial with durvalumab [52], and CORINTH trial with pembrolizumab (NCT04046133) (Table 1).

## 4. How to Move Forward beyond DCF in Advanced SCCA

### 4.1. SCCA and Associated Antigen

HPV-associated cancers are an interesting model to study antigen-specific T-cell responses. Indeed, HPV16 E6 and E7 proteins are implicated in the carcinogenesis of this disease. It has been shown that E6 and E7 specific CD4 and CD8 T-cell responses were detected in the peripheral blood of patients with high-grade anal squamous intraepithelial lesion and may be associated with spontaneous regression [53]. Moreover, HPV16 E6 protein activates transcription of hTert reverse-transcriptase gene [54]. The reactivation of hTert, an enzyme overexpressed in more than 90% of human cancers, induces immortalization of cancer cells [55]. In Epitopes HPV01 and HPV02 trials, SCCA-related specific immune responses were measured by ELISpot assays using CD8 and CD4 T-cell recognizing E6 and E7 peptides and telomerase promiscuous peptides presented in most HLA-DR contexts to specifically monitor antigen-specific TH1 cells [56,57]. The data showed that DCF chemotherapy increased the frequency of IFNγ producing lymphocytes [24]. Only anti-hTert Th1 CD4 T-cell responses measured after DCF chemotherapy could predict the probability of progression free survival at 12 months (62.5% of patients with post-DCF anti-hTert TH1 immunity were free of progression at 12 months compared to 23.5% hTert non-immunological responders, ***p*** = 0.0175) [10]. These results support the use of hTert as an HPV-related antigen in SCCA. Furthermore, the prognostic value of telomerase CD4 Th1 immunity measured after DCF chemotherapy raises the hypothesis that adaptive immune responses promoted by DCF chemotherapy contribute to the duration of the clinical efficacy observed in these studies. Recently, it has also been reported the prognostic value of CD8 infiltrating T-cells in SCCA patients [17,58]. In these studies, the presence of CD8 infiltrating T-cells was associated with good tumor differentiation, early-stage diagnosis and better PFS. Moreover, patients responding to the immunotherapy had higher percentage of CD8 infiltrating T-cells in tumor samples at baseline in comparison with nonresponder patients. Thus, the presence of CD4 Th1 immunity or CD8 infiltrating T-cells can be an interesting biomarker to predict survival and efficacy of chemotherapies and immunotherapies treatments in SCCA patients.

### 4.2. M-MDSC, an Interesting Biomarker in SCCA Patients

MDSC represents a very heterogeneous population of immature myeloid cells and is critical in the regulation of immune responses by suppressing the function of antigen presenting cells and inhibit antigen-specific T-cell responses [59]. In addition, MDSC boosts the tumor microenvironment by promoting expansion and immunosuppressive functions of regulatory T-cell and tumor-associated-macrophages. MDSC represents a promising biomarker that correlates with the clinical outcome in several solid tumors [60,61].

The prognosis value of Monocytic-MDSC (M-MDSC) in SCCA patients treated by DCF chemotherapies was observed in two clinical trials [24]. Indeed, after DCF chemotherapy, the percentage of M-MDSC was diminished among SCCA patients and permitted to select a threshold (1.2%) for further analyses. Moreover, high M-MDSC levels were significantly predictive of a shorter PFS at baseline (14.6 vs. 11.0 months, *p* = 0.044) and after DCF chemotherapy (26.7 vs. 11.0 months, *p* = 0.0083). Similar results were observed with OS. Interestingly, no change in monocyte or Treg levels was observed compared to healthy donors, and there was no correlation either with OS nor PFS before or after DCF treatment, thus sustaining the specific impact of M-MDSC on SCCA patients’ survival.

Furthermore, the percentages of patients with peripheral lymphocytes responding to E6, E7, and hTert had decreased in the presence of high M-MDSC levels. Strikingly, DCF selectively enhanced the frequency and the intensity of hTert Th1 immune responses only when M-MDSC levels were below 1.2%. These data show that high-risk M-MDSC population inhibits antigen-specific T-cell responses in SCCA patients. Interestingly, 8 out of 13 patients (61.5%) with hTert Th1^high^ and M-MDSC^low^ immune profile measured after DCF were free of progression compared to 3 out of 12 patients (25%) with hTert Th1^low^ and M-MDSC^high^ immune profile. No similar correlation could be established for E6 and E7 responses, suggesting that hTert CD4 Th1 immune responses and M-MDSC levels are biomarkers particularly interesting in SCCA disease.

Thus, these results emphasized the key role of hTert-specific CD4^+^ Th1 responses and M-MDSC levels as prognostic factors to better stratify SCCA patients’ risk of death.

### 4.3. Angiogenesis and MDSC: An Interesting Pathway in SCCA Patients

The immunosuppressive activities of MDSC depend on the activation of several signaling pathways such as PI3K/AKT and RAS/MAPK signaling and their transcription factors such as HIF-1α and Signal transducer and activator of transcription (STAT) proteins [62]. It has been previously reported that Angiopoietin-2 (Ang2) and STAT3 are involved in pejorative clinical outcomes in cancer patients [63,64,65]. Indeed, STAT3 plays a key role in the activation of MDSC and is strongly implicated in MDSC expansion and immunosuppressive functions [66].

**Figure 1:**
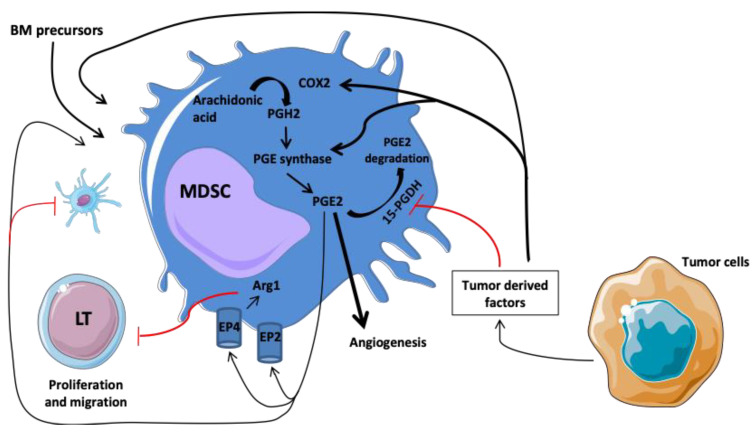
Involvement of COX2/PGE2 pathway in the regulation and differentiation of MDSC. The tumor microenvironment promotes the production of COX2 promoting the synthesis of PGE2. EP4 receptor expressed on MDSC can bind PGE2 and induced arginase-1 (Arg1) production. Arg1 accelerates l-arginine consumption, resulting in suppression of T-cell proliferation. Abbreviations: Arg1, arginase; BM, bone marrow; and PGH2, prostaglandin H2.

Prostaglandin E2 (PGE2) acts as a mediator of inflammatory signaling to promote angiogenesis to prevent dendritic cell maturation and to allow the immunosuppressive functions of endothelial cells and myeloid cells. As a matter of fact, PGE2 might contribute to the immunosuppressive functions of MDSC during cancer progression. Indeed, the EP4 receptor expressed on MDSC can bind PGE2 and induce arginase-1 (Arg1) production [67]. Arg1 accelerates l-arginine consumption, resulting in the suppression of T-cell proliferation by several mechanisms such as reduction of IFNγ/IL-2 secretion from T cells and CD3 ζ-chain expression (Figure 1) [68].

Ang2 is a proangiogenic and immunomodulatory factor in late stages of cancer development. The Tie2 receptor can bind Ang2 and is constitutively expressed on some monocytes [69]. In NSCLC patients, the activated Ang2/Tie2+ M-MDSC axis inhibits T-cell responses directed against (TAAs). Interestingly, the addition of recombinant Ang2 during in vitro stimulation abolished IFNγ production in response to TAAs. By contrast, the elimination of Tie2-expressing M-MDSC before in vitro stimulation either restored or significantly increased tumor-associated-antigens T-cell responses in 6 of 13 patients untreated metastatic NSCLC [70]. These data identify the Ang2high/Tie2high M-MDSC axis as a participant in tumor immune evasion that should be considered in future cancer immunotherapy.

All of these data raised the hypothesis that M-MDSC subsets might be a critical biomarker to predict clinical outcomes and PD-L1 efficacy in SCCA patients.

## 5. Advanced Disease: Time for Immunotherapy-Based Combinations

Docetaxel is a microtubule-stabilizing agent, and exerts cytotoxic functions by blocking dividing cells in G2/M phase, leading to apoptosis [71]. Docetaxel can also induce calreticulin, a damage-associated molecular pattern related to the immunogenic cell death [72]. Another observation indicates that docetaxel might influence the inhibition of immunosuppressive cells, sustaining the potential restoration of an effective anti-tumor immunity [73]. In patients with advanced SCCA, DCF was capable of enhancing HPV-adaptive immune responses and decreasing immunosuppressive cells in peripheral blood, sustaining its development in association with an anti-PD1/L1 immunotherapy. To date, three randomized clinical trials are ongoing to evaluate the efficacy of a taxane-based chemotherapy with an anti-PD1/L1. The SCARCE phase II trial with mDCF+/−atezolizumab has finished its recruitment, and the final results are pending [21]. Moreover, POD1UM-303 double-blinded phase III trial with CP in combination with retifanlimab or placebo is ongoing (NCT04472429), as well as an NCI open-label phase III trial with CP+/−nivolumab (NCT04444921).

In second line and further, anti-PD1/L1 immunotherapy is the best evidence-based treatment. Five prospective trials included 298 patients. ORR was observed in 40 patients (13.4%) including 11 patients (3.7%) with a complete response. The median OS was around 11 months [17,18,19,20,74]. Despite the efficacy of this treatment, the great majority of chemorefractory patients do not benefit from an anti-PD1 monotherapy. Then, several combinations were evaluated. Atezolizumab was associated to bevacizumab in a “basket” trial, with no sign of a synergic effect. Among 19 evaluable patients with SCCA, two patients (11%) presented an objective response, and the median OS was 11.6 months. Hence, the addition of an antiangiogenic limited to VEGF/VEGFR axis does not seem to be interesting in SCCA. One hypothesis could be the potential antiangiogenic effect of the protein p16 [75] expressed in almost all SCCA [76]. CARACAS “pick the winner” phase II randomized study evaluated avelumab vs. avelumab plus cetuximab. ORR was higher with the combination (17%) compared to avelumab alone (10%). However, it seems that there is probably an additive effect more than a synergic one with no difference in long-term outcomes [74]. The best combination results come from the association of an anti-PD1/L1 with a tumor vaccine. ISA101 is an HPV16 E6 and E7 protein-targeting vaccine and was combined with nivolumab in a phase II trial in HPV16+ SCC of different origin. Among 24 patients, 8 patients (33%) presented an objective response, including two (8.3%) complete responses. The median OS was 17.5 months [77]. INO-3112, a plasmid DNA vaccine, which comprises three plasmids expressing HPV-16 and HPV-18 E6 and E7 proteins along with IL-12, was associated with induction of HPV-16/18 E6/E7–specific immune responses and showed a synergistic activity with anti-PD1 therapy in HPV+ head and neck cancer patients [78]. INO-3112 in combination with durvalumab is ongoing in patients with recurrent or metastatic HPV+ cancers including SCCA (NCT03439085). Interestingly, in Epitopes-HPV01 and Epitopes-HPV02 studies, a significantly better prognosis was associated with hTERT immunity and not with anti-HPV-E6/E7 immunity, suggesting that hTERT vaccines are promising in SCC patients, in combination with an anti-PD1/L1. Currently, VolaTIL phase II trial is ongoing with an hTERT vaccine (UCPVax) [79] in combination with atezolizumab, in HPV+ squamous cell carcinomas including SCCA (NCT03946358) (Table 1).

## 6. Monitoring Circulating HPV Tumor DNA in SCCA

Modified DCF is the most effective (complete response rate, 40.3%) with the best safety profile (grade 3/4 toxicity rate, 53%) treatment among combined chemotherapy regimens in first line and became standard in advanced SCCA [10,16]. Interestingly, the conversion from positive to negative HPV ctDNA on liquid biopsy, which is highly predictive of better prognosis, was achieved in 61.1% (22/36) of patients with DCF in the Epitopes-HPV02 trial, compared to 17.9% (5/28) of patients with doublet chemotherapy in InterAACT trial [11,16,23]. In consequence, CP regimen should be considered as an option in patients with a contraindication to 5FU or cisplatin (e.g., impaired renal function and active cardiovascular disease) [16,80].

The detection of circulating tumor DNA (ctDNA) in plasma has become an important biomarker in recent years for the diagnosis of theragnostic mutations (e.g., EGFR and lung cancer). It has been shown in many cancers that the monitoring of ctDNA level is strongly associated with tumor response and can predict tumor relapse before conventional imaging [81,82]. The advantages of ctDNA over more traditional blood biomarkers, such as SCC antigen, are a very short blood half-life (~few hours) and a specificity of almost 100%. As SCCA is secondary to HPV infection in the vast majority of cases (mainly HPV16) and HPV is present in the tumor cell either in episomal form or integrated into the DNA [83], it is also possible to detect HPV circulating tumor DNA (HPV ctDNA) [82,84,85,86] using either NGS [86] or digital-PCR techniques [23,87]. It has been shown that the specificity is also very high, and that HPV ctDNA is not detected in microinvasive cervical lesions [85]. The advantages of HPV ctDNA detection compared to "classical" ctDNA (e.g KRAS/TP53 mutation) are: (i) The presence of HPV in >90% of cases and the low cost of tumor HPV genotyping (while testing for tumor genomic alterations can be negative and is more expensive). (ii) The higher detection sensitivity of HPV ctDNA. Indeed, when a mutation (e.g., PIK3CA) is usually present only at an average of one copy/tumor cell, tumor HPV DNA is present at 100–1000 copies/tumor cell, thereby increasing the amount of ctDNA releases in plasma after cell death.

Several studies have investigated the monitoring of HPV ctDNA in SCCA at different stages of cancer, and have shown that: (i) HPV ctDNA detection is possible for different genotypes with a very good sensitivity and is associated with tumor stage, (ii) HPV ctDNA usually becomes undetectable during radio-chemotherapy, and post-radio-chemotherapy detection of HPV ctDNA is significantly associated with a very poor prognosis, (iii) post chemotherapy HPV ctDNA detection at metastatic stage is associated with a poor progression free survival, and HPV ctDNA monitoring is possible during immunotherapy. Presently, we do not know which technique (ddPCR vs NGS) is more sensitive. While Lee et al suggested in a study of 21 patients that NGS-based approach had an almost perfect sensitivity, even in small tumor (T1/T2) [86], it has to be noted that NGS is more time consuming and expensive. A large prospective study (Circa HPV NCT03739775) is ongoing in patients with HPV-induced cancer and will evaluate the clinical validity of this biomarker during treatment and follow-up. However, despite all these studies, the clinical utility of HPV ctDNA to improve patient care still need to be evaluated in clinical trials. In an INTERACT-ION neoadjuvant trial in stage III SCCA, HPV ctDNA detection will be considered after the induction phase for further CRT in SCCA.

## 7. Conclusions

This is an exciting moment in clinical research in SCCA. Historical barriers regarding this disease are vanishing thanks to the research networks in this rare pathology, gained knowledge in tumor biology and its environment, and the arrival of immunotherapies. A better present is possible thanks to the taxane-based chemotherapy in advanced disease, and the future is promising as well with novel immunotherapy combinations.

## Figures and Tables

**Table 1:** Immunotherapy combination clinical trials.

Localized Disease			Advanced Disease		Treatment	Trial Number	Phase
Neoadjuvant	Concomitant	Adjuvant	First-Line	≥Second-Line			
		NCI-EA2165			Nivolumab + IMRT	NCT03233711	III
INTERACT-ION					Ezabenlimab + mDCF + IMRT	NCT04719988	II
	RADIANCE				Durvalumab + IMRT	NCT04230759	II
	CORINTH				Pembrolizumab + IMRT	NCT04046133	I/II
	BrUOG 276				ADXS11-001 + IMRT	NCT01671488	I/II
			POD1UM-303		Retifanlimab + CP	NCT04472429	III
			NCI-EA2176		Nivolumab + CP	NCT04444921	III
			SCARCE		Atezolizumab + mDCF	NCT03519295	II
			SPARTANA		Spartalizumab + mDCF + SBRT	NCT04894370	I/II
				VolaTIL	Atezolizumab + UCPVax	NCT03946358	II
				NCI-2015-01004	Nivolumab + ISA101	NCT02426892	II
				NCI-2018-00914	Durvalumab + INO311	NCT03439085	II
				NCI-20-C-0104	M7824 + PRGN-2009	NCT04432597	I/II
				NCI9673	Nivolumab + Ipilimumab	NCT02314169	II
				DUET-2	XmAb20717	NCT03517488	I
				HESTIA	Nivolumab + HPVST cells	NCT02379520	I
				CARACAS	Avelumab + Cetuximab	NCT03944252	II
				NCI-2017-00501	Atezolizumab + Bevacizumab	NCT03074513	II

Abbreviations: mDCF, modified docetaxel, cisplatin, 5-fluorouracil regimen; IMRT, intensity-modulated radiation therapy; CP, carboplatin and paclitaxel regimen; SBRT, stereotactic body radiation therapy; UCPVax, universal cancer peptide CD4 telomerase vaccine; ADXS11-001, ISA101, INO3112 and PRGN-2009 are HPV anti-tumor vaccines; M7824, anti PD-L1 mAb fused with 2 extracellular domains of TGF-βRII; XmAb20717, bispecific anti-PD-1/anti-CTLA-4 mAb; HPVST cells, adoptive HPV+ tumor-directed T-cells.

**Table 2:** Clinical trials in locally advanced disease.

Trial	Phase	Stage	RT Dose	Induction CT	Concomitant Treatment	Results	Ref.
ACCORD 03	III	T ≥ 4 cm or N+	60 Gy vs. 70 Gy	CDDP + 5FU	CDDP + 5FU	Negative	[7]
RTOG 98-11	III	≥T2	45–59 Gy	CDDP + 5FU	CDDP + 5FU or MMC + 5FU	Negative	[6]
ACCORD 16	II	T ≥ 3 cm or N+	65 Gy	-	CDDP + 5FU + cetuximab	Stop for toxicity	[48]
ECOG 3205	II	65% stage III	45–54 Gy	CDDP + 5FU	CDDP + 5FU + cetuximab	Negative	[49]
FFCD 0904	II	T ≥ 2 cm or N+	65 Gy	-	MMC + 5FU + panitumumab	Negative	[46]

Abbreviations: RT, radiotherapy; CT, chemotherapy; CDDP, cisplatin; 5FU, 5-fluorouracil; MMC, mitomycin.
